# Plasma triglyceride levels and central obesity predict the development of kidney injury in Chinese community older adults

**DOI:** 10.1080/0886022X.2019.1655451

**Published:** 2019-10-10

**Authors:** Yujie Cao, Guangshan Sun, Rui Liu, Ao Sun, Qian Zhang, Yang Li, Lele Wang, Xiangli Chao, Xiaojie Zhou, Sha Zhang, Ruping Chen

**Affiliations:** a Graduate School of Tianjin Medical University, Tianjin, People’s Republic of China;; b Department of Clinical Laboratory, Tianjin Union Medical Center, Tianjin, People’s Republic of China;; c Tianjin Junliangcheng Hospital, Tianjin, People's Republic of China;; d Tianjin Children's Hospital, Tianjin, People's Republic of China

**Keywords:** Kidney injury, chronic kidney disease (CKD), glomerular filtration rate (GFR), triglyceride, central obesity, elderly population

## Abstract

**Objective:** Despite the achievement of blood glucose, blood pressure targets, the risk for kidney injury remains high among older adults. This observational retrospective study investigated whether high TG or high WC contribute to this high residual risk for kidney injury.

**Methods:** A total of 843 elderly from Dongli Community, Tianjin, China, we selected 666 individuals with a baseline estimated glomerular filtration rate (eGFR) ≥60 mL/min/1.73 m^2^ and negative microalbuminuria completing a 3-year follow-up. At baseline, subjects were grouped according to the levels of TG and WC. The primary outcome was the incidence of kidney injury, defined as low eGFR (eGFR <60 mL/min/1.73 m^2^) or reduced eGFR (eGFR reduced >25%) or UACR ≥30 mg/g.

**Results:** Overall, 6.01% developed low eGFR, 11.11% reduced eGFR, 25.98% UACR ≥30 mg/g, and 3.45% low eGFR and UACR ≥30mg/g after 3-year follow-up. TG ≥1.7 mmol/L increased the risk of eGFR <60 mL/min/1.73 m^2^ by 1.44-fold, of UACR ≥30 mg/g by 32%, and of developing both abnormality by 1.41-fold in model 1; further adjustment for potential confounders factors, the association is slightly weakened in model 2 and 3; WC (≥90 cm in men and ≥85 cm in women) were associated with a 1.68-fold higher risk of eGFR <60 mL/min/1.73 m^2^ and a 1.43-fold risk of UACR ≥30 mg/g and a 1.89-fold risk of developing both abnormality in model 1. Further adjustment for potential confounders factors, the association is slightly weakened in model 2 and 3.

**Conclusions:** In a population of Chinese community-dwelling older adults, high TG and central obesity were risk factors for the development of kidney injury over 3 years.

## Introduction

Kidney dysfunction is rising worldwide in parallel with population ageing and chronic kidney disease (CKD) is more common in older people [1]. It is not only a precursor of end-stage renal disease (ESRD), but also an important risk factor of cardiovascular disease (CVD), even in the early stage of renal impairment [2]. According to Kidney Disease: Improving Global Outcomes (K/DOQI) Work Group, CKD is interpreted as a structural or functional kidney abnormality lasting for 3 or more months [3]. The National Health and Nutrition Examination Survey showed that the prevalence of kidney injury is 38% among those aged >65 years, compared with 13% of the overall US population [4]. Similarly, a survey from Chinese general population, the prevalence of CKD among females increases from 7.4% among those aged 18–39 years to 18.0 and 24.2% among those aged 60–69 and 70 years, respectively [5]. As the prevalence of kidney injury has increased globally along with an aging population, it has become a major health problem incurring substantial healthcare costs [4].

Kidney injury is associated with developing metabolic syndrome (MetS). The number of patients with MetS is increasing and has become a risk factor for the development of kidney injury, therefore, predicting the development of CKD patients [6,7]. It is estimated that the MetS affects at least a quarter of the adult population worldwide [8]. In American adults, the prevalence of MetS increases with age, reaching 18.3% for people aged 20–39 and 46.7% for people over 60 [9]. In China, the overall prevalence of men is 19.2% and the prevalence of women is 27.0%, which is also higher in older groups [10]. Metabolic syndrome is a group of metabolic disorders centered on insulin resistance and basic characteristics include abnormal glucose metabolism, hypertension, central obesity, disorder of lipid metabolism [11]. At present, there is no universally recognized standard for diagnosing metabolic syndrome in the world and there are great differences in different regions. The diagnostic criteria for MetS in China are proposed in the 2017 China guidelines for the prevention and treatment of type 2 diabetes, (1) central obesity (WC ≥90 cm in men and ≥85 cm in women); (2) hypertriglyceridemia: fasting plasma TG ≥1.7 mmol/L; (3) elevated blood pressure: BP ≥130/85 mmHg, antihypertensive drug treatment in a patient with a history of hypertension is an alternate indicator; (4) hyperglycemia: fasting glucose level of ≥6.1 mmol/L, drug treatment of elevated glucose is an alternate indicator; (5) decreased HDL-C: fasting HDL-C<1.04 mmol/L. These risk factors can be isolated or intertwined to increase the severity of chronic kidney disease. Wong et al. [12] have shown that intensive glycemic control is associated with long-term development of ESKD and its benefits are in retaining renal function. The relationship between hypertension and the occurrence of adverse vascular events, including progression of kidney injury, is clear and independent of other confounding factors [13].

Although the increasing prevalence of kidney damage in older individuals seems to be partly because of an increasing prevalence of hyperglycemia and hypertension [14]. However, in spite of the achievement of recommended targets for blood glucose and blood pressure, the residual risk for kidney injury remains high. Additional components of the MetS-high triglyceride (TG) and high waist circumference (WC)-may be the factors responsible for this high residual risk [3]. TG are heritable risk factors for vascular disease [15], and more recently, it has been suggested that high TG is shown to accelerate the rate of renal damage [16]. It is reported that TG levels begin to increase in the early stage of kidney injury and reach a peak in CKD stage IV. Obesity also seems to increase the risk of developing major risk factors for CKD. Among the US population with CKD, the prevalence of obesity is even higher, at 44.1% [17]. The traditional definition of obesity is based on body mass index (BMI). However, obesity can be further assessed in terms of fat distribution via WC and WC is a measure of central obesity that reflects metabolically active visceral fat [18]. Therefore, high TG and central obesity have a certain impact on kidney injury, which may lead to a high residual risk of kidney injury. This article needs to further explore the effects of high TG and central obesity on elderly kidney function.

## Materials and methods

### Study participants

This was a retrospective observational study who subjects followed up at Dongli community, Tianjin, China participating in a physical examination. The analysis was performed using a data set of electronic medical records collected between 2011 and 2013. For the purpose of the analysis, we considered only individuals who were ≥60 years old and had at 3 years follow-up for data. The last visit with complete renal data was considered the 3-year evaluation. Among the total of 843 subjects identified, we excluded those with microalbuminuria positive, eGFR<60 mL/min/1.73 m^2^, urine positive RBC or WBC and those with missing data at baseline. A total of 666 subjects met the inclusion criteria and were included in the study.

### Measurements

All TG and WC had been measured according to diagnostic criteria for MetS in Chinese guidelines for the prevention and treatment of type 2 diabetes in 2017. All samples were taken and measured in the clinical laboratory. The determination of TG and serum creatinine was performed on Abbott IC16000 and manufacturer provided reagents and had been standardized. The eGFR was evaluated using CKD-EPI (Chronic Kidney Disease Epidemiology Collaboration) formula, which is based on serum creatinine concentrations and age [19]. The quantification of urinary albumin was assessed by nephelometry using the Dade–Behring BNII special protein analyzer and manufacturer-provided reagents. Urinary creatinine was measured using the enzymatic method with the ABBOTT ARCHITECT C16000. ACR was then calculated. Smoking was defined as one or more cigarettes per day, including cessation within the past 3 months. Drinking was defined as 50 mL or more per day, including cessation within the past 3 months. The primary outcomes were (1) low eGFR(eGFR <60 mL/min/1.73 m^2^); (2) reduced eGFR (eGFR reduced >25%); (3) UACR ≥30 mg/g; (4) low eGFR and UACR ≥30mg/g. Low eGFR is defined as eGFR <60 mL/min/1.73 m^2^, and reduced eGFR is defined as eGFR reduced >25%. We only involved chronic kidney injury, CKD. The diagnosis of CKD is based on the criteria of the 2012 Kidney Disease: Improving Global Outcomes, that is, a single measurement showing eGFR <60 mL/min/1.73 m^2^ or proteinuria (albumin to creatinine ratio (ACR) ≥30 mg/g) or both [3].

The study was approved by the Health Bureau of Dongli District of Tianjin and all participants gave written informed consent. The study approval number was 2011-C01.

### Statistical analysis

Baseline clinical and biochemical characteristics are presented as mean values ± standard deviation (SD), categorical variables are described as frequencies and percentages. The difference between groups was tested using the Chi-squared test for categorical variables. The main analysis aimed to evaluate the association between baseline TG and WC with renal outcomes during the study period. Associations of renal outcomes with risk factors, including TG and WC were expressed as an odds ratio (OR), which was calculated using logistic regression analyses. Analysis was built regression models based on the cognizance of different set of confounders, such as, model 1 adjusted for age, sex; model 2 adjusted for age, sex, TG, WC, BMI, SBP, DBP, FBG, TC; model 3 adjusted for above + lipid-lowering treatment, blood-pressure-lowering treatment, antidiabetic treatment. Analyses were performed by using SPSS 20 (IBM Corporation, Armonk, NY, USA) and GraphPad Prism 7 (GraphPad Software, Inc., La Jolla, CA, USA). *p* Values <.05 were considered statistically significant.

## Results

### Baseline characteristics of study subjects stratified by TG and WC values

Table 1 shows the baseline clinical characteristics of participants with subjects stratified by TG and WC values. As expected from the study design, renal function was preserved at baseline, with a mean eGFR of 83.79 mL/min/1.73 m^2^. Overall, most clinical data for subjects with or without high TG concentrations (≥1.7 mmol/l) showed small but significant differences. Subjects with high TG concentrations at baseline had a higher BMI (26.39 vs. 24.34 kg/m^2^) and WC (92.02 vs. 86.63 cm), slightly worse glucose control with higher FBG. Subjects in the high TG group were more frequently female and more use of lipid-lowering medications (67.95 vs. 3.24%) and had more hypertension or diabetes. Also, the percentages of the group who were smokers and who were receiving antihypertensive treatments and antidiabetic treatment were larger in the high TG group. Similar findings were observed when comparing the baseline characteristics of subjects with normal and high WC values (Table 1). Subjects with high WC had a higher BMI, worse glucose control, a higher hypertension or diabetes, a higher percentage of subjects taking lipid-lowering, antihypertensive medications and antidiabetic treatment. However, no differences were noted in basal use of antidiabetic medications when stratifying the study population.

**Table 1.** Baseline characteristics of study subjects stratified by TG and WC values.

		TG ≥1.7 mmol/l			WC ≥90 cm(men) or ≥85 cm(women)		
	ALL (*n* = 666)	No (*n* = 432)	Yes (*n* = 234)	*p*	No (*n* = 276)	Yes (*n* = 390)	*p*
Male sex (%)	279 (41.89%)	202 (46.76%)	77 (32.91%)	.001	126 (45.65%)	153 (39.23%)	.098
Age (year)	65.58 ± 5.40	66.42 ± 5.62	64.03 ± 4.61	<.001	66.26 ± 5.61	65.10 ± 5.21	.006
SBP (mmHg)	133.64 ± 18.85	132.09 ± 18.70	136.50 ± 18.83	.004	130.07 ± 16.69	136.17 ± 19.87	<.001
DBP (mmHg)	85.06 ± 9.23	84.48 ± 8.92	86.14 ± 9.71	.026	83.55 ± 8.55	86.13 ± 9.56	<.001
TC (mmol/l)	5.17 ± 2.18	5.03 ± 2.55	5.40 ± 1.20	.023	5.22 ± 3.18	5.14 ± 1.98	.616
TG (mmol/l)	1.69 ± 1.10	1.10 ± 0.32	2.79 ± 1.19	<.001	1.31 ± 0.67	1.96 ± 1.26	<.001
BMI (kg/m²)	25.06 ± 3.48	24.34 ± 3.49	26.39 ± 3.05	<.001	22.73 ± 2.88	26.71 ± 2.86	<.001
WC (cm)	88.65 ± 9.36	86.63 ± 9.37	92.02 ± 8.36	<.001	80.04 ± 5.80	94.74 ± 6.01	<.001
FBG (mmol/l)	5.18 ± 1.46	5.10 ± 1.42	5.33 ± 1.53	.053	5.07 ± 1.42	5.26 ± 1.49	.089
Scr (mmol/l)	71.97 ± 10.45	71.79 ± 10.13	72.31 ± 11.03	.541	71.22 ± 10.02	72.51 ± 10.72	.116
Urea nitrogen (mmol/l)	6.02 ± 2.13	6.04 ± 1.56	5.98 ± 2.92	.742	5.96 ± 1.59	6.06 ± 2.45	.569
Smoking (%)	287 (43.09%)	180 (41.67%)	107 (45.73%)	.312	138 (50%)	149 (38.21%)	.002
Hypertension (%)	344 (51.65%)	197 (45.60%)	147 (62.82%)	<.001	116 (42.03%)	228 (58.46%)	<.001
Diabetes (%)	69 (10.36%)	37 (8.56%)	32 (13.68%)	.039	22 (7.97%)	47 (12.05%)	.089
Drinking (%)	132 (19.82%)	100 (23.15%)	32 (13.68%)	.003	57 (20.65%)	75 (19.23%)	.65
Lipid-lowering treatment (%)	173 (25.98%)	14 (3.24%)	159 (67.95%)	<.001	21 (7.61%)	152 (38.97%)	<.001
Blood-pressure-lowering treatment (%)	345 (51.80%)	194 (44.91%)	144 (61.54%)	<.001	111 (40.22%)	227 (58.21%)	<.001
Antidiabetic treatment (%)	71 (10.66%)	34 (7.87%)	32 (13.68%)	.017	21 (7.61%)	45 (11.54%)	.095
eGFR (ml/min/1.73 m²)	83.79 ± 10.46	84.26 ± 10.33	82.93 ± 10.65	.115	84.70 ± 10.46	83.16 ± 10.42	.061

Data are mean ± SD or absolute frequency (percentage).

eGFR: estimated glomerular filtration rate; ACR: albumin-to-creatinine ratio; SBP: systolic blood pressure; DBP: diastolic blood pressure; BMI: body mass index; TG: triglyceride; TC: total cholesterol; FBG: fasting blood-glucose; WC: waist circumference; Scr: serum creatinine.

### Clinical characteristics by renal outcome at 3 years of follow-up

**Figure 1.**
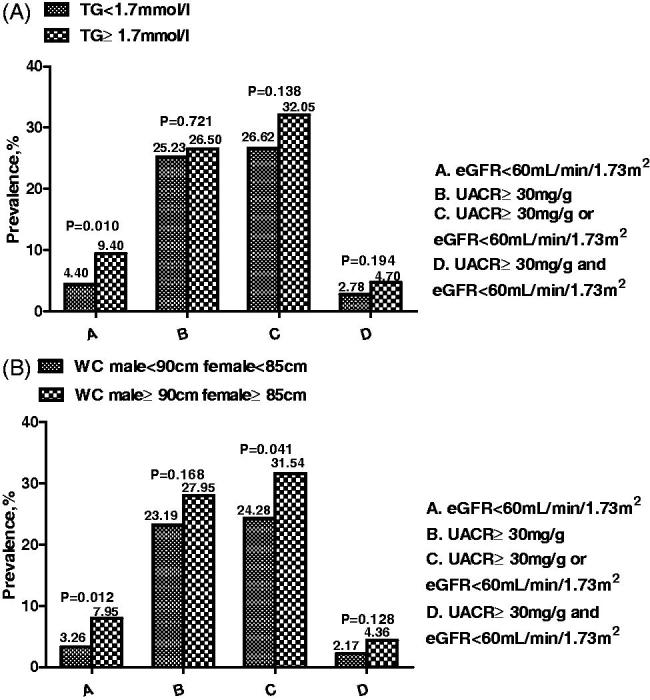
(A) Renal outcomes incidence according to baseline TG ≥1.7 mol/L. (B) Renal outcomes incidence according to baseline WC (≥90 cm in men; ≥85 cm in women).

At the end of the 3 years of follow-up, among 666 study subjects, 40 (6.01%) developed low eGFR values, 74 (11.11%) showed reduced eGFR, 173 (25.98%) developed UACR ≥30 mg/g, and 23 (3.45%) developed both low eGFR and UACR ≥30 mg/g (Table 2). As shown in Figure 1, all renal outcomes were significantly worse in subjects with high TG (Figure 1(A)) and/or high WC (Figure 1(B)) values than in subjects with baseline TG and WC values in the normal range.

**Table 2.** Renal outcomes in the study population at the 3-year follow-up.

	Study participants *n*(%)
eGFR <60 ml/min/1.73 m² or UACR ≥30 mg/g	190 (28.53%)
eGFR <60ml/min/1.73 m² and UACR ≥30 mg/g	23 (3.45%)
eGFR <60 ml/min/1.73 m²	40 (6.01%)
UACR ≥30 mg/g	23 (13.29%)
UACR <30 mg/g	17 (3.45%)
eGFR reduction >25% than baseline	74 (11.11%)
eGFR <60 ml/min/1.73 m²	36 (90%)
eGFR ≥60 ml/min/1.73 m²	38 (6.07%)
UACR ≥30 mg/g	33 (19.08%)
UACR <30 mg/g	41 (8.32%)
UACR ≥30 mg/g	173 (25.98%)
eGFR <60 ml/min/1.73 m^2^	23 (57.50%)
eGFR ≥60 ml/min/1.73 m^2^	150 (23.97%)

Table 3 shows baseline characteristics according to the development of renal outcomes after 3-year follow up. Subjects developing low eGFR within 3 years of follow-up (*n* = 40) were more frequently female and older. They also had higher baseline blood pressure(BP), FBG, TG and higher hypertension or diabetes. Similar findings were noted when comparing baseline variables of patients who developed UACR ≥30 mg/g (*n* = 173) at follow-up versus those who remained UACR<30 mg/g, with the exception of the percentage of drinkers, which was larger among those developing UACR ≥30 mg/g.

**Table 3.** Baseline clinical characteristics by renal outcome after 3 years.

	eGFR ≥60 ml/min/1.73 m^2^ (626)	eGFR<60 ml/min/1.73 m^2^ (40)	*p*	UACR <30 mg/g (493)	UACR ≥30 mg/g (173)	*p*
Male sex (%)	266 (42.49%)	13 (32.5%)	.214	220 (44.62%)	59 (34.10%)	.016
Age (year)	67.20 ± 5.23	71.05 ± 6.90	<.001	67.16 ± 5.22	68.21 ± 5.90	.028
SBP (mmHg)	131.60 ± 19.50	138.25 ± 20.15	.037	130.18 ± 18.24	137.18 ± 22.26	<.001
DBP (mmHg)	82.40 ± 12.09	83.13 ± 15.45	.718	82.00 ± 12.65	83.71 ± 11.22	.115
TC (mmol/l)	5.25 ± 2.84	5.31 ± 1.38	.898	5.14 ± 1.02	5.20 ± 1.02	.503
TG (mmol/l)	2.28 ± 6.84	3.92 ± 12.25	.167	2.02 ± 5.14	1.82 ± 1.13	.61
BMI (kg/m²)	25.62 ± 6.61	27.50 ± 11.03	.098	25.30 ± 5.48	25.50 ± 3.52	.66
WC (cm)	86.54 ± 11.55	85.58 ± 16.53	.618	86.23 ± 12.03	87.22 ± 11.52	.347
FBG (mmol/l)	6.25 ± 11.52	8.72 ± 22.61	.225	6.50 ± 13.43	6.12 ± 9.14	.732
Scr (mmol/l)	76.62 ± 10.25	102.57 ± 16.36	<.001	77.86 ± 11.62	79.12 ± 14.31	.252
Urea Nitrogen (mmol/l)	5.86 ± 2.67	7.94 ± 2.91	<.001	6.01 ± 3.04	5.93 ± 1.50	.769
Smoking (%)	275 (43.93%)	19 (47.50%)	.666	232 (47.06%)	62 (35.84%)	.011
Drinking (%)	149 (23.80%)	6 (15%)	0.202	119 (24.14%)	36 (20.81%)	.373
Hypertension (%)	361 (57.67%)	36 (90%)	<.001	280 (56.80%)	117 (67.63%)	.012
Diabetes(%)	83 (13.26%)	9 (22.50%)	.101	57 (11.56%)	35 (20.23%)	.004
Lipid-lowering treatment (%)	228 (36.42%)	21 (52.5%)	.042	183 (37.12%)	65 (37.57%)	.916
Blood-pressure-lowering treatment (%)	362 (57.83%)	37 (92.50%)	<.001	280 (56.80%)	117 (67.63%)	.012
Antidiabetic treatment (%)	82 (13.10%)	9 (22.50%)	.093	57 (11.56%)	34 (19.65%)	.008
eGFR (ml/min/1.73 m²)	78.94 ± 9.09	53.28 ± 6.49	<.001	78.25 ± 10.31	74.94 ± 11.93	.001

Data are mean ± SD or absolute frequency (percentage).

eGFR: estimated glomerular filtration rate; ACR: albumin-to-creatinine ratio; SBP: systolic blood pressure; DBP: diastolic blood pressure; BMI: body mass index; TG: triglyceride; TC: total cholesterol; FBG: fasting blood-glucose; WC: waist circumference; Scr: serum creatinine.

### Regression models associations of baseline TG and WC levels and renal outcomes at follow-up

Table 4 shows regression models associations of high TG and high WC with renal outcomes at follow-up. TG >1.7 mmol/L and WC ≥ 90 cm(men) or ≥80 cm (women) were associated with eGFR <60 mL/min/1.73 m^2^ (OR2.44 and 2.68) in model one. Further adjustment for factors which were potential confounders and unlikely to be in the causal pathway between the high TG and high WC and eGFR <60 mL/min/1.73 m^2^ had an impact on the odd ratios, high TG and high WC were still significantly associated with eGFR <60 mL/min/1.73 m^2^. The ORs for eGFR<60 mL/min/1.73 m^2^ were 2.01 and 2.66 in model two. When adjusted for lipid-lowering treatment, blood-pressure-lowering treatment and antidiabetic treatment, the association of high TG and high WC and eGFR <60 mL/min/1.73 m^2^ were slightly weakened (OR 1.71 and 2.48) in model three. High TG and high WC were associated with UACR ≥30 mg/g (OR 1.32 and 2.43) in model one. Further adjustment for potential confounders factors, high TG and high WC were associated with UACR ≥30 mg/g is slightly weakened, the ORs for UACR ≥30 mg/g were 1.12 and 1.59 in model two. When adjusted for lipid-lowering treatment, blood-pressure-lowering treatment and antidiabetic treatment, the association between high TG and high WC and UACR ≥30 mg/g is still slightly weakened in model three. The associations between high TG, WC and eGFR <60 mL/min/1.73 m^2^ and UACR ≥30 mg/g were true.

**Table 4.** Associations between baseline TG and WC and renal outcomes after 3-year followup.

	Model 1		Model 2		Model 3	
	OR (95% CI)	*p*	OR (95% CI)	*p*	OR (95% CI)	*p*
eGFR <60 ml/min/1.73 m²						
TG ≥1.7 mmol/l	2.44 (1.27,4.68)	.007	2.01 (1.00,4.04)	.05	1.71 (0.72,4.05)	.221
WC ≥90 cm (men) or ≥85 cm (women)	2.68 (1.25,5.75)	.012	2.66 (1.08,6.58)	.034	2.48 (0.98,6.31)	.057
UACR ≥30 mg/g						
TG ≥1.7 mmol/l	1.32 (0.56,3.11)	.528	1.12 (0.78,1.60)	.552	0.71 (0.23,2.26)	.566
WC ≥90 cm (men) or ≥85 cm (women)	2.43 (0.95,6.19)	.063	1.59 (0.54,4.70)	.406	1.37 (0.45,4.18)	.579
eGFR<60 ml/min/1.73 m² and UACR ≥30 mg/g						
TG ≥1.7 mmol/l	2.41 (1.28,4.56)	.007	1.88 (0.95,3.72)	.068	1.63 (0.69,3.81)	.263
WC ≥90 cm (men) or ≥85 cm (women)	2.89 (1.35,6.17)	.006	2.53 (1.03,6.19)	.042	2.35 (0.93,5.95)	.07

Model 1: Adjusted for age, sex.

Model 2: Adjusted for age, sex, TG, WC, BMI, SBP, DBP, FBG, TC.

Model 3: Adjusted for above + lipid-lowering treatment, blood-pressure-lowering treatment, antidiabetic treatment.

### Renal outcome risk associated with high TG and high WC levels according to age, sex, and common risk factors

**Figure 2.**
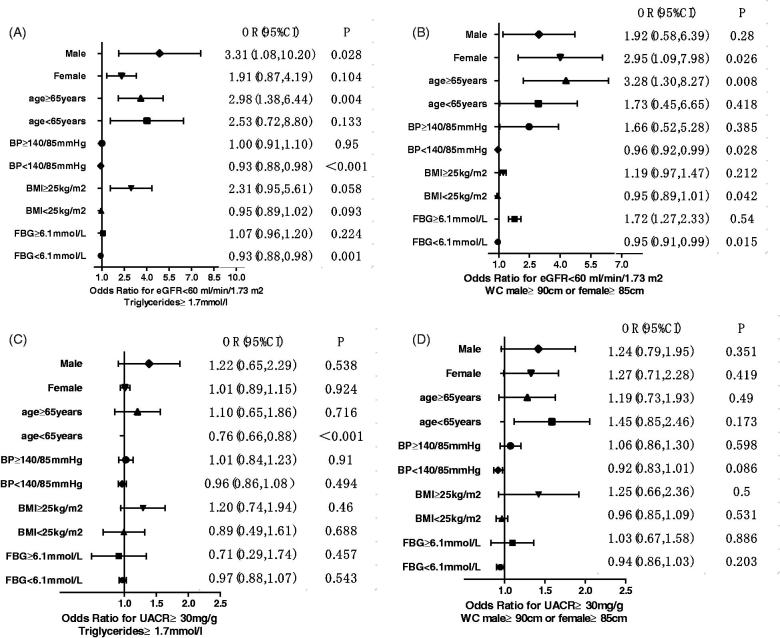
Multivariate associations of high TG and WC levels with renal outcomes after stratification for sex, age, and several risk factors. Data are shown as ORs with 95% CI for eGFR<60 mL/min/1.73 m^2^ (A and B) and for albuminuria (C and D).

As shown in Figure 2, high TG and/or high WC levels significantly increased the risk of developing renal outcomes after factoring for sex, age, blood pressure, BMI, FBG levels.

## Discussion

Although the incidence of kidney damage is gradually increasing among the elderly, the therapeutic progress has led to a larger number of subjects reaching the recommended targets for blood glucose and blood pressure, which can further reduce the burden on the kidneys. However, in addition to the known factors affecting kidney injury, there may be other confounding factors for its residual risk. Therefore, our longitudinal cohort study aimed to investigate the effects of high TG and central obesity on renal function in elderly subjects with preserved renal function in the north China community. In short, our objective is to more effectively find out the elderly kidney injury patients.

In our cohort of 666 subjects with normal kidney at baseline, 6.01% developed low eGFR values, 11.11% showed reduced eGFR, 25.98% developed UACR ≥30 mg/g and 3.45% developed both low eGFR and UACR ≥30 mg/g. This incidence is lower than the previous study by Russo et al. [20], they reported that 12.8% developed low eGFR, 7.6% showed reduced eGFR, 23.2% albuminuria, and 4% albuminuria and either low eGFR or an eGFR reduction >30%. This may depend on selection bias, their enrolled population is diabetic, but we chose elderly who participated in community hospital physical examinations and who were healthy people and also combined with various diseases such as diabetes and hypertension.

Lipoprotein abnormalities have been identified as possible causes of kidney damage, especially glomerular injury [21]. Dyslipidemia is associated with nephropathy and has been shown to play a key role in the development and progression of kidney disease. The mechanism behind this effect has not been fully elucidated, but it has been suggested that lipids may damage the blood vessels of the kidney, mesangial cells and tubular cells. Moorhead et al. first proposed the “lipid nephrotoxicity hypothesis.” [22] High TG is one of the clinical components of the MetS and may be a consequence of the underlying kidney damage. Furthermore, the TG to HDL-C ratio is a better marker for identifying renal insufficiency [23]. Our data also clearly indicate high TG levels are important risk factors for the development of kidney and the adjusted risk for developing any renal event associated with higher TG levels was still higher. Although it seems that the risk of glomerular injury is slightly lower than the risk of decreased renal function. These associations were partly attenuated by multivariate adjustment. Lipid-lowering drugs used by the elderly in our study included fibrates and statins. Antihypertensive therapy, including oral antihypertensive drugs such as nifedipine, amlodipine, compound reserpine. Antidiabetic treatment, including oral hypoglycemic drugs, such as acarbose, metformin or subcutaneous injection of insulin. Although the fibrates, statins, acarbose and metformin might have damaged the kidneys, they are only contraindicated in patients with severe renal impairment. All subjects had a normal renal function at the start of the study and they were dosed strictly in accordance with the drug instructions. Therefore, there was no potential confusion about medical treatment. Recent systematic reviews concluded that lipid-lowering therapy decreases death events in patients with CKD [24]. For example, statins might be more potent to block the development of kidney disease [25]. The target range of dyslipidemia in cases of CKD, however, remains to be determined and there may be individual differences. Thus, evaluation and the target range of treatment of dyslipidemia should be individualized.

Obesity has become a worldwide epidemic and its prevalence has been projected to grow by 40% in the next decade. This increasing prevalence has implications for the risk of CKD [26]. Chen et al. [27] showed that increased WC was significantly related to microalbuminuria and reduced GFR, suggesting that central obesity might be an independent risk for kidney injury, which is in agreement with our data. However, Huh et al. [28] demonstrated that there was no significant association between abdominal obesity and kidney injury, this finding is inconsistent with our study. There are different versions of the diagnostic criteria for MetS and they consider that a possible explanation for this discrepancy is that the cutoff values of obesity for renal damage may be different from those of MetS. At present, there are some differences in the study of the effects of WC on kidney damage. Therefore, we need to explore this issue further.

Age is a major determinant of outcomes in kidney injury. Older patients with CKD are much more likely to die of complications related to aging and CVD before developing ESRD [29]. A review report that kidney function declines with age, people over the age of 40 experience a loss of GFR of about 1 mL/min/year and accelerates slightly in the later years. This steady decline occurs in the context of widespread age-related tissue damage seen in all organs of the body [30]. TG and WC are affected by many factors, while the elderly have more basic diseases, which have a great impact on the two. Just like our researchers, there are many subjects with hypertension and diabetes, and taking antihypertensive and hypoglycemic drugs, these underlying diseases may cause high WC and TG or mutual influence. Since the subject of this article is the elderly in northern China, most people in northern China have halophilic, which may be one of the reasons leading to higher hypertension. It is reported that long-term adherence to the Dietary Approaches to Stop Hypertension (DASH)-style diet may contribute to the prevention of CKD [31]. Moreover, sex may be a factor that potentially influences the association of kidney injury risk with dyslipidemia and central obesity. Many studies have shown that women have a higher prevalence of CKD than men. This simplest explanation is that the longer life expectancy of women combined with the natural decline of kidney function with aging contributes to an enlarged population at risk for CKD [29]. Furthermore, women's WC are higher than men's, which also causes the prevalence of women's kidney injury to be higher. In our study, women with high WC had a higher risk of kidney damage when low eGFR was a dependent variable.

Limitations of our present study should be considered when interpreting these results. First, the sample size of our study was relatively small, especially only 666 participants were included. Second, the electronic medical records reflect the patient's previous physical health status, such as the past history of the disease and family history, which has some guiding significance for the study, which may affect the assessment of clinical risk. Thirdly, positive urine protein only one test, multiple measurements are required to average. Finally, regarding dyslipidemia, we lack indicators such as HDL-C, which may not be perfect. However, we are longitudinal studies that allow us to infer causality between the high TG, WC and kidney injury risk.

## Conclusion

In a population of Chinese community-dwelling older adults, high TG levels and central obesity were risk factors for the development of kidney injury over 3 years. Treatment of risk factors would help slow down the reduction of kidney function in elderly patients. At last, regular health check or selective screening of the elderly population is an effective method for early detection of kidney injury.

## Abbreviations

CKD: chronic kidney disease
eGFR: estimated glomerular filtration rate
UACR: urinary albumin to creatinine ratio
TG: triglyceride
WC: waist circumference
Lipid-lowering treatment: oral lipid-lowering, such as statins and fibrates
Blood pressure-lowering treatment: oral antihypertensive drugs, such as nifedipine
Antidiabetic treatment: oral hypoglycemic agents or subcutaneous insulin injections
